# Hair Loss: Evidence to Thallium Poisoning

**DOI:** 10.1155/2018/1313096

**Published:** 2018-06-26

**Authors:** Guifang Yang, Changluo Li, Yong Long, Lijuan Sheng

**Affiliations:** ^1^Department of Emergency, The Second Xiangya Hospital, Central South University, China; ^2^Department of Emergency, The Changsha Central Hospital, Changsha, China; ^3^Department of Neurology, The Second Xiangya Hospital, Central South University, China

## Abstract

In clinical practice, thallium poisoning is very hard to diagnose, because it is a very uncommon disease and its clinical manifestations are extremely complicated. In the present study, we investigated a case of a 53-year-old man who was hospitalized for persistent stabbing pain in the abdomen and lower extremities for 20 days. Physical examination revealed diffuse alopecia of the scalp. The final diagnosis of thallium poisoning was confirmed based on high blood and urine thallium levels. The patient was cured by an oral administration of Prussian blue combined with hemoperfusion and continuous veno-venous hemofiltration.

## 1. Introduction

The colorless, odorless, and tasteless thallium is often reported to be used nefariously and illegally, because of its slow-acting presence and its ability to induce a wide range of symptoms. Early signs of poisoning usually begin with pain without any obvious causes, which can be easily misleading to other illnesses and conditions [1]. Thallium poisoning is rarely encountered in emergency departments. Excessive exposure to thallium may occur in many ways, such as the maintenance and cleaning of the ducts and flues by smelters and overdosing on drugs such as cocaine and heroin. Criminal and unintentional thallium poisoning cases have also been reported, some of which have led to death [2]. From 1995 to 2005, the American Association of Poison Control Centers reported 830 cases of human exposures to thallium, and one death from 1995 to 2009 [3] was included. Because of the low incidence rate and nonspecific clinical manifestation of thallium poisoning, in diagnosing practice, the disease was hardly considered by physicians [4]. In this study, a 53-year-old man with thallium poisoning was misdiagnosed on several occasions before being successfully treated. In the following 6 months of follow-up, there was no repetitive poisoning present or sequela. Finally, this case demonstrates the entire process of hair loss following thallium poisoning.

## 2. Case Report

**Figure 1:**
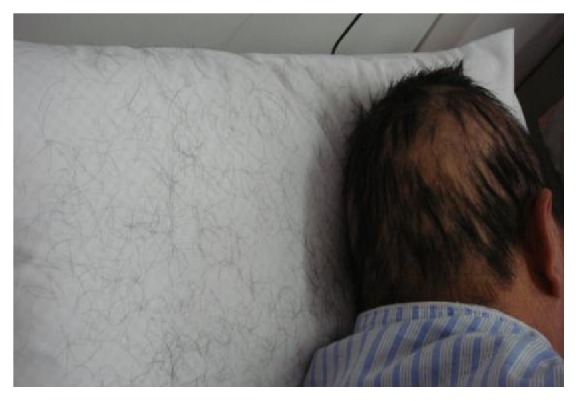
Hair partial loss (February 20, 2014).

**Figure 2:**
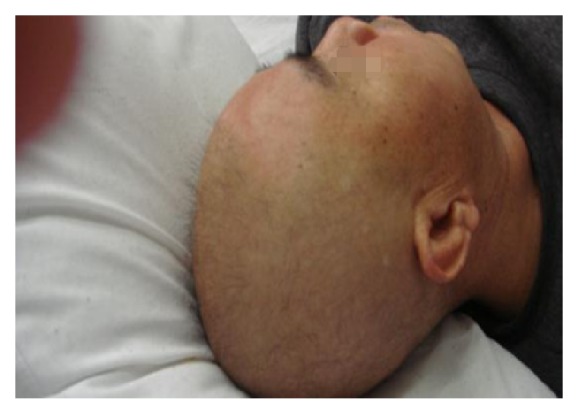
All hair loss (February 26, 2014).

**Figure 3:**
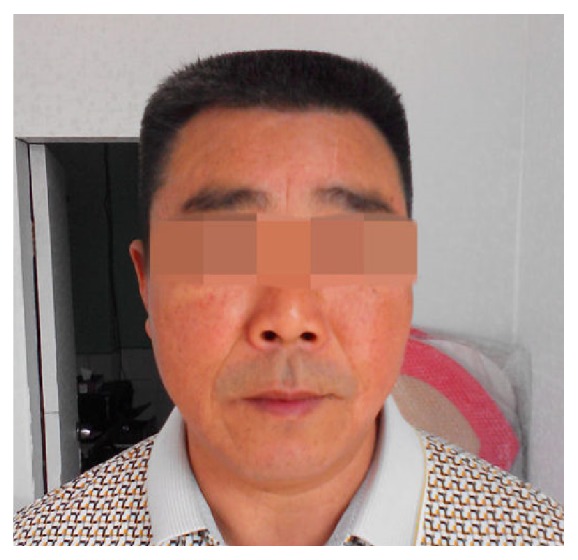
Hair grown completely (September, 2014).

A 53-year-old man was locally hospitalized for persistent aching pain in the abdomen and lower extremities for nearly 20 days. He was sent to our clinic because of acute and severe hair loss which occurred for 10 days. According to his self-reported history, he was healthy in the past and did not take any medications. He also denied the possibility of accidental poisoning. Physical examination revealed diffuse alopecia of scalp (Figure 1). His liver function was damaged (ALT 154.8u/l and AST 49.2 u/l), but the levels of urine mercury, hair arsenic, and blood lead were all normal. The scalp hair was completely lost (Figure 2) 1 week later. The diagnosis of thallium poisoning gradually came to our mind and was eventually confirmed by the elevated levels of the thallium ion in urine (4677.0*μ*g/l, normal range 5*μ*g/l) and blood (312.1*μ*g/l, normal range 0*μ*g/l). Therefore, ten cycles of hemoperfusion and hemodialysis were performed, each lasting for 5 to 6 hours daily. As hemoperfusion and hemodialysis were conducted, the blood and urine thallium levels decreased. After hemoperfusion and hemodialysis, there was improvement in neurological manifestations and liver function parameters. At the same time, he was given Prussian blue 2.64 g (0.33 g *∗* 8), Q6h, forced diuresis and 20 mmol potassium chloride twice a day, and intravenous B complex. His pain disappeared slowly, and hair regrowth started 10 days later and was completed during his 6-month follow-up (Figure 3). The reason for poisoning remains unknown despite an investigation conducted by police authorities.

## 3. Discussion

Thallium is a silver white heavy metal which was first discovered by William Crookes [5]. In the past, thallium was used as a therapeutic agent to treat syphilis, gonorrhea, tuberculosis, and ringworm, and it was also used as a depilatory for excess hair. The mortality of the acute thallium poisoning is about 6% to 15%. The main reason of death is respiratory failure. Meanwhile, about 33% to 50% patients of survivors have sequelae such as nervous system diseases and visual impairment [6].

The main toxicological effect of thallium is as follows: (1) it can interfere with a series of enzymes whose activation depends on potassium function. For example, pyruvate kinase, an enzyme, can affect the glucose metabolism. In addition, thallium is thought to have a tenfold higher affinity to sodium–potassium ATPase compared with potassium, thus interrupting its binding activity [7]. (2) Thallium also appears to bind to sulfhydryl groups located on the mitochondrial membrane, hence interfering with its normal functions. This is illustrated by the acute hair loss, which could have been caused by thallium's ability to bind to cysteine sulfhydryl groups found in hair. Moreover, thallium binds to glutathione which inhibits its activation and the inability to metabolize heavy metals which causes their overaccumulation in the body [8]. (3) Thallium could change cell membrane properties of the liposome, which impacts related enzyme activity and the material transshipment [9]. The major clinical manifestation of thallium poisoning includes gastrointestinal reaction and multiple peripheral neuropathy. It is also characterized by hair loss [10], which usually occurs within 2-3 weeks after the poisoning [11]. In the six months of follow-up, our case demonstrated the whole process of the hair change after thallium poisoning. Prompt diagnosis of thallium poisoning is difficult. For example, the patient in our study was only able to be diagnosed properly one month after poisoning. To date, the diagnosis of thallium poisoning mainly depends on the patients' history of contact poison and the laboratory results of blood thallium or urine thallium. Due to the fact that the victims usually have very limited awareness about their consumption or exposure to the poisoning substance, the characteristic symptoms of thallium poisoning are imperative for diagnosis [4]. As in the present study, we still have no idea how the patient was poisoned. In the end, we made our diagnosis of acute thallium poisoning by referring to the blood (>100ug/L) and urine (>200ug/L) thallium levels [12].

In the past, the specific antidote to thallium poisoning was still unknown, which was changed in 2003, and the United States Food and Drug Administration (FDA) approved Prussian blue as a promising antidote candidate. Currently, it is recommended that the treatment by Prussian blue should be orally administrated 250 mg/(kg · d) 4 times a day. The drug should be dissolved into 15% or 20% mannitol (50 ml). Although both Prussian blue and activated charcoal absorb thallium, it appears that Prussian blue has absorptive superiority. In addition, because it has a far better safety profile than other proposed therapies, Prussian blue should be considered as the priority of the drug in managing acute thallium poisoning [13]. At the same time, the patient may take magnesium sulfate orally to promote excretion of thallium [14]. Forced diuresis with potassium loading was previously recommended to increase the renal clearance of thallium [14]. Due to the small molecular size and low binding affinity to protein, thallium is thought to be dialyzable. Therefore, hemoperfusion and hemodialysis should be conducted early in massive toxicities [15]. In our study, the patient received significant clinical improvement of neurological manifestations and liver function parameters after ten cycles of hemoperfusion and hemodialysis, which was in consistent with the findings revealed in Misra's [16] and Lu's [17] researches. This indicated that both hemoperfusion and hemodialysis could play an effective role in eliminating thallium.

In recent years, an increasing number of thallium poisoning cases were reported in China; one important reason may be that the supervisions on the sales, usage, and managements of thallium-included salt have not drawn much attention from our government. After analyzing studies in China, we found that the sources of thallium in a considerate number of thallium poisoning cases were from the online purchases of thallium compound. Therefore, we thought it is urgent for government to limit thallium in market to prevent thallium crime.
